# Obliteration of radical cavities with autogenous cortical bone; long-term results

**DOI:** 10.1186/1472-6815-8-4

**Published:** 2008-07-29

**Authors:** Akram M Abdel-Rahman, Matti Pietola, Teemu J Kinnari, Hans Ramsay, Jussi Jero, Antti A Aarnisalo

**Affiliations:** 1 Audiology Unit, Dept. of Otorhinolaryngology, Mansoura Univ., Mansoura, Egypt; 2 Dept. of Otorhinolaryngology, Univ. of Helsinki, Helsinki, Finland

## Abstract

**Background:**

To evaluate the long-term surgical outcome(s) in patients who have undergone canal-wall-down operation with mastoid and epitympanic obliteration using autologous cortical bone chips, bone pate and meatally-based musculoperiosteal flap technique.

**Method:**

Retrospective evaluation of seventy patients operated during 1986–1991 due to a cholesteatoma. An otomicroscopy was performed to evaluate the postoperative outer ear canal configuration with a modified Likert scale (1 – 4). The outer ear canal physical volume was assessed by tympanometry. The hearing outcome and a patient-filled questionnaire were also analyzed.

**Results:**

The posterior wall results were 1.8 (± 0.9 SD) and the attic region 1.8 (± 0.9 SD) (ns., p > 0.05). These values show either no cavity formation or minor formation of a cavity, with a good functional result. The mean volume of the operated ear canal was 1.7 (± 0.5 SD) ml. The volume of the contralateral ear canal was 1.2 (± 0.3 SD) ml (*** p < 0.0001). A comparison of the current mean ABG to the preoperative mean ABG and to the ABG at one-year postoperatively, 5-years postoperatively or 10-years postoperatively showed no statistical significance (p > 0.05).

**Conclusion:**

ABG does not significantly change in the long-term. The configuration of the cavity tends to change, however, the obliteration material is stable in the long-term and clinically significant cavitation rarely occurs.

## Background

Canal-wall-down (CWD) tympanomastoidectomy is a well established method in surgery due to cholesteatoma [1]. It provides a good intraoperative exposure and an easy postoperative monitoring. The size of the surgical cavity can be diminished with obliteration to create a small cavity that is self-cleaning and easily maintained [2]. Several authors have demonstrated the usefulness of mastoid obliteration technique and considered it a safe method to diminish a surgical cavity in CWD surgery [3-7]. However, there are only a few studies that have evaluated the configuration of the cavity and the durability of the obliteration material itself in the long-term [6,8-10].

CWD surgery creates a large open cavity, with several possible problems [11]. Both autologous and synthetic materials have been used for obliteration [3,12-14]. In our clinic, autologous cortical bone chips and pate, a meatally based musculoperiosteal flap ("Palva flap"), and temporal fascia are mainly used. The CWD tympanomastoidectomy with this obliteration method has been well characterized [15,16].

We have studied the long term condition of ears that underwent CWD mastoidectomy with mastoid and epitympanic obliteration using autogenous bone chips, bone pate and a musculoperiosteal flap. All the patients that participated in the study were followed at least for 15 years. Clinical experience suggests that the configuration of the cavity changes during the long-term follow-up. Parts of the cavities tend to enlarge and cavities that are difficult to treat occur [17]. We have measured the volumes of the reconstructed ear canals, and evaluated the configuration of a possible cavity and problems caused by that.

## Methods

### Patients

Between the years 1986 – 1991, a series of 133 CWD tympanomastoidectomies, due to a cholesteatoma, were performed. A musculoperiosteal flap ("Palva flap") was used with autogenous bone for obliteration and reconstruction, by experienced otosurgeons at the Department of Otorhinolaryngology, University of Helsinki. The autogenous cortical bone chips (approximate size 0.5 – 1 cm^2^), and cortical bone pate were taken from the temporal bone, above the mastoid. The mastoid cavity and epitympanum were filled with these bone chips and bone pate. The obliterated cavity was lined with a piece of temporal fascia and with the musculoperiosteal flap. The flap was not fixed. The ossicular chain was reconstructed either during the analyzed CWD surgery with obliteration or in a later tympanoplasty with autologous ossicular bone or autologous cortical bone. Meatoplasty was not performed routinely. The ear was packed and at the one week follow-up, the packing was removed.

All these patients were invited to the study and were sent an informed consent. Of the 94 patients willing to participate, 70 took part in the study. The patient data, operative details and audiological evaluations were collected retrospectively and analyzed.

### Ethical consideration

The design of this study was proved by the local ethical committee at the Department of Otorhinolaryngology, University of Helsinki. All these patients gave their written informed consent to participate in the study.

### Otological and audiological examination

An otomicroscopy and a tympanometry were performed. In addition, the diameter of the entrance of the outer ear canal was measured with the aid of an ear speculum (6 mm, 8 mm, 10 mm or more). The formation of the cavity in the outer ear canal and in the attic region was evaluated with a modified Likert scale (1 = no cavity formation; 2 = minor formation of a cavity, with a good functional result; 3 = moderate cavity formation, crusting; 4 = major cavity formation, crusting, cholesteatomatic growth in the cavity) by four otosurgeons. Pure-tone audiometry thresholds (0.5, 1, 2 and 4 kHz PTA) via air- (AC) and bone-conduction (BC) were determined and the air-bone gap (ABG) was calculated. The patients filled in a questionnaire and the data of the questionnaires were analyzed with PrismStat (GraphPad Software Inc., San Diego, USA) (Appendix 1). In the questionnaire the patients were asked the frequency of mastoid debridements and whether the ear had been dry or had it discharged since the surgery. Also problems with the use of hearing aids were evaluated. The t-test was used for statistical analysis (* p < 0.05).

## Results

### Patient population

In our study there was a male preponderance, 48 males versus 22 females participated. The mean age of the patients at the operation was 40 (± 13 SD) years (range from 7 to 66 years) and at the time of evaluation was 59 (± 13 SD) years (median 60 years) with a mean follow-up period 18 (± 1.5 SD) years (median 18 years).

Twenty patients had been previously (before 1986–1991 period) operated due to a cholesteatoma (Table 1). Of those patients, three had been operated three times and one patient operated twice. Nearly all the previous operations had been CWD operations, without obliteration or reconstruction of the attic. Of the patients we evaluated between 1986 and 1991 all were aimed to be single stage surgeries. During the analyzed CWD surgery, six patients had a meatoplasty. After the CWD surgery, 21 (30%) had a second operation to the evaluated operated ear (Table 2). One patient was operated twice and one three times. Six patients were operated due to a recurrent cholesteatoma (9%). No residual cholesteatomas were found in our material.

**Table 1:** Operations to the studied ear prior the evaluated CWD surgery

Type of surgery	Number of surgeries
CWD operation, without obliteration	19 (27%)
Simple mastoidectomy	4 (6%)
CWU operation	3 (4%)
Atticotomy	1 (1%)

The type and number of ear surgeries to the analyzed ear prior to the analyzed operation (20 patients).

**Table 2:** Operations to the studied ear after the evaluated CWD surgery

Type of surgery	Number of surgeries
Meatoplasty	9 (13%)
Myringoplasty	2 (3%)
Tympanoplasty	7 (10%)
Re-radical operation	6 (9%)

The type and number of ear surgeries of patients that had revision surgeries to the analyzed ear after the analyzed operation (21 patients).

### Otomicroscopy and volume of the cavity

In 32 patients we had to use a 6 mm ear speculum. In 29 patients it was possible to use 8 mm ear speculum and only in four cases a 10 mm speculum or larger was used. The size and configuration of the ear canal and/or cavity were estimated. The formation of a cavity in the operated ear was evaluated with an otomicroscope (modified Likert scale, 1 – 4). Most of the operated ears showed a good functional result. The posterior wall of the ear canal and the attic region were analyzed separately. The posterior wall results were 1.8 (± 0.9 SD) and the attic region 1.8 (± 0.9 SD) (ns. p > 0.05) (Table 3). One tympanic membrane perforation was seen. An aerated tympanum was found in 52 patients and an adhesive tympanum was found in 18 patients. The mean volume of the operated ear canal was 1.7 (± 0.5 SD) ml (n = 70). The volume of the contralateral ear canal was 1.2 (± 0.3 SD) ml (n = 59)(*** p < 0.0001).

**Table 3:** Posterior wall and attic reconstruction

Mean	1	2	3	4
1.8 ± 0.9 (posterior wall)	28 (40%)	29 (41%)	7 (10%)	5 (7%)
1.8 ± 0.9 (attic)	31 (44%)	25 (36%)	9 (13%)	4 (6%)

The degree of cavitation was analyzed with a modified Likert scale (1 = no cavity formation; 2 = minor formation of a cavity, with a good functional result; 3 = moderate cavity formation, crusting; 4 = major cavity formation, crusting, cholesteatomatic growth in the cavity). Posterior wall and attic region were analyzed separately. The numbers of patients with different degrees of cavitation are shown. The mean of cavitation values are shown on the left. (ns., p > 0.05).

### Audiological data

The mean AC, BC and ABG values (value ± SD) at different time points are shown in Table 4. A significant difference could be seen between the current mean AC values compared to all other time points (pre-operative, p = 0.002; 1-year, p = 0.001; 5-years, p = 0.004; 10-years, 0.004). Also the current mean BC values were significantly different compared to all the other time points (pre-operative, p < 0.001; 1-year, p < 0.001; 5-years, p = 0.01; 10-years, p < 0.001). However, no significant differences could be seen between the current mean ABG and other time points (pre-operative, p = 0.9; 1-year, p = 0.7; 5-years, p = 0.09; 10-years, 0.6). Twenty patients (29%) had an excellent (0–10 dB) or a good (11–20 dB) gap closure one-year after the evaluated surgery. Currently 25 patients (36%) have an excellent or good gap closure in the operated ear (Table 5).

**Table 4:** Mean AC (air conduction), BC (bone conduction) and ABG (air-bone gap) values of the patients during the follow-up

	Preoperative	Postoperative 1-yr	Postoperative 5-yr	Postoperative 10-yr	Postoperative current
Mean AC	47 ± 5	47 ± 9	45 ± 8	45 ± 7	59 ± 9
Mean BC	21 ± 7	21 ± 8	24 ± 7	20 ± 5	31 ± 9
Mean ABG	27 ± 7	25 ± 6	22 ± 5	25 ± 7	26 ± 7

The preoperative, 1-year postoperatively, 5-years postoperatively, 10-years postoperatively mean AC, BC and ABG values (all 0.5, 1, 2 and 4 kHz PTA)(value ± SD).

**Table 5:** Patients air-bone gaps during the follow-up

Air-bone gap (dB)	Preoperative		Postoperative 1-yr		Postoperative 5-yr		Postoperative 10-yr		Postoperative current	
	No.	%	No.	%	No.	%	No.	%	No.	%

0–10 (excellent)	4	6	5	8	5	11	5	15	4	6
11–20 (good)	16	24	20	31	15	41	11	32	21	30
21–30 (fair)	20	29	14	5	10	27	7	21	19	27
> 30 (poor)	28	43	25	39	7	19	11	32	26	37

The preoperative, 1-year postoperatively, 5-years postoperatively, 10-years postoperatively and the current ABG of the operated ear.

### Questionnaire

**Figure 1:**
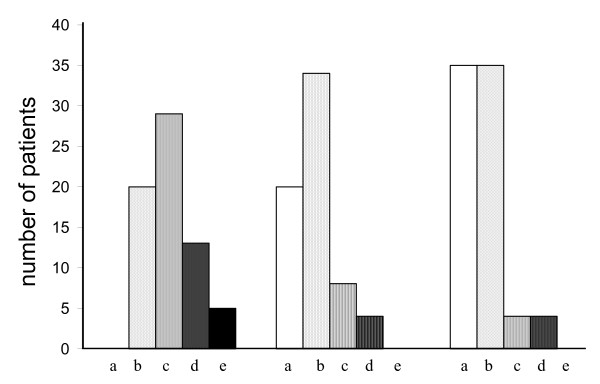
**A histogram showing the need of cavity debridement.** On the left side: 1–2 years after the surgery (a = no need for debridement, b = once per year or less, c = 1–2 times/year, d = 3–6 times/year, e = over 6 times/year); on the middle set of bars; 5–10 years after the operation; on the right hand side; current situation.

The need for debridement of the cavity was evaluated. Currently, 35 patients (50%) had no need for debridement of the cavity. The need for debridement diminished in the long run (Figure 1).

Twenty-nine patients (40%) were hearing aid users. Twenty-two of these (76%) were using a hearing aid in the operated side or in both sides. Sixteen of these patients (73%) were comfortable using the hearing aid in the operated ear. On the other hand, five patients had recurrent problems and four patients were unable to wear the aid in the operated ear. Typical problems which these patients were encountering: recurrent infection of the cavity due to hearing aid use (7 patients), fitting problems (5 patients), poor hearing level (6 patients) or combination of these.

## Discussion

A total of 133 patients underwent CWD surgery with complete epitympanic and mastoid obliteration 18 (± 1.5 SD) years ago at our department. Of these, 94 patients were willing to participate and 70 (53%) took part in the study. The data of patients that gave their written informed consent but did not actually participate, were not included. Dense cortical bone chips, collected from the mastoid bone, and cortical bone pate were used for the obliteration of the surgical cavity. A meatally based musculoperiosteal flap was raised when CWD surgery was anticipated.

We were especially interested in the stability of the obliteration material that was used. The musculoperiosteal flap atrophies postoperatively, but it often leaves a smooth lining for the cavity [15]. Reconstructed canal walls and reconstructed epitympanic cavities tend to enlarge postoperatively [2]. Sometimes these pockets are deep and may cause cholesteatomatic growth in the cavity.

Our results show that the mastoid obliteration with autogenous cortical bone, mainly cortical bone chips, is an efficient and long lasting way to diminish the surgical cavity. Most of the cavities were small and predominantly trouble free. The meatally based musculoperiostel flap will provide a smooth surface for the cavity and deep pockets with cholesteatomatic growth are rare. We observed no significant difference in the resorption of bone from the epitympanic area compared to the mastoid area.

No meatoplasty was routinely done to our patients during the analyzed CWD surgery with obliteration. However, in six cases meatoplasty was performed. During the follow-up, meatoplasty was done to nine patients. Twenty patients were operated earlier and some of them in other hospitals. It is possible that some of these patients had meatoplasty, which was not mentioned in our patient records. To our knowledge, at least 15 patients (21%) had a meatoplasty. During the last follow-up visit, it was possible to do an otomicroscopy to all of the patients and reliably evaluate the ear canal and tympanic membrane.

The acoustic properties of an obliterated cavity are considered to be better than an open cavity [18-21]. It is possible that new bone formation facilitated by a periosteal flap in the CWD cavity may provide acoustic properties similar to those of the normal ear canal [18,22,23]. Our results with hearing were acceptable, showing that in CWD surgery, the tympanoplasty does save the hearing even in the long run. The differences between the preoperative, 1-year, 5-year, 10-year and current ABGs were not statistically significant. 25 patients (36%) showed good or excellent gap closure after a mean follow-up period of 18 ± 1.5 years. This can be considered as a good result.

Twenty-nine patients (40%) were hearing aid users. In most cases the aid was fitted into the operated ear. Those patients felt comfortable using the hearing aid in the operated ear. The benefit of using a hearing aid among our patients seems to be the same as with elderly unoperated patients [24,25]. Also the problems our patients encountered were of same kind as compared to elderly unoperated hearing aid users [24,25].

A 7-year follow-up of the reconstructed ear canals showed that the ear canal has a tendency to enlarge due to the atrophy of the musculoperiosteal flap [15,16]. Our results show that the difference of the volume between the operated and unoperated ears is significant. However, clinically this difference is still quite unsignificant due to the fact that most of the cavities were self-cleaning and did not contain significant retractions.

Epidermoid cells that are not removed at the initial operation can regrow and cause a residual cholesteatoma [11]. Even beyond 6 years follow-up residual cholesteatomas are still observed [11]. No residual cholesteatomas were seen in our material during a mean follow-up period of 18 ± 1.5 years. No cholesteatomas were found or operated that would have been located among the obliteration material. The autogenous material was always taken before the actual surgery.

Formation of new retraction pockets to the cavity can retain keratin and develop into a new disease as a recurrent cholesteatoma. Six revision radical operations (9%) were performed after the initial CWD surgery. These operations were done due to recurrent cholesteatoma and dehiscence of the autogenous obliteration material. An attempt was made to perform the CWD operations with obliteration as a single stage surgery, but revisions were required in 21 patients (30%) with six exhibiting recurrent cholesteatoma (9%).

## Conclusion

Currently, over half of our patients do not need regular debridements. The configuration of the cavity tends to change in the long-term. However, the obliteration material is stable and clinically significant cavitation rarely occurs.

## Competing interests

The authors declare that they have no competing interests.

## Authors' contributions

AMA–R took part in the design of the study, acquisition, analysis and interpretation of data and drafting of the manuscript. MP participated in the acquisition of data, the analysis and interpretation of data and drafting of the manuscript. TJK took part in acquisition, analysis and interpretation of data. HR and JJ took part to the acquisition, analysis and interpretation of data and both revised the manuscript critically for important intellectual content. AAA took part in the design of the study, acquisition, analysis and interpretation of data and drafted the manuscript. All the authors have read and approved the final version of the manuscript.

## Appendix 1

The questionnaire complited by the patients

The Questionnaire

1. How often did/do you visit your surgeon due to problems related to the operated ear?

a) 1–2 years after the surgery

1 once/year or seldom

2 1–2 times/year

3 3–6 times/year

4 over 6 times/year

b) 5–10 years after the surgery

1 not at all

2 once/year or seldom

3 1–2 times/year

4 3–6 times/year

5 over 6 times/year

c)lately

1 not at all

2 once per year or seldom

3 1–2 times/year

4 3–6 times/year

5 more than 6 times/year

2. Has the ear discharged after the surgery?

1 never

2 there have been only a few periods of discharge

3 the ear is discharging frequently

4 it is discharging continuously

3. Have you had a revision operation? Yes no

When? Where?

4. Are you using a hearing aid? Yes no

5. I use hearing aid(s):

1 in the operated ear

2 in the contralateral ear

3 in both ears

6. Are you comfortable with using a hearing aid?

1 no problems

2 occasional problems

3 often difficult

4 due to problems I do not wear it in the operated ear

7. What kinds of problems did/do occur if you are using a hearing aid?

1 ear starts to discharge

2 insertion problems with the ear piece

3 the hearing level of the ear is too poor to wear an aid

4 others? What kinds of problems?

8. How long have you been using a hearing aid?

1 1–2 years

2 3–6 years

3 7–10 years

4 over 10 years

## Pre-publication history

The pre-publication history for this paper can be accessed here:


